# Synthesis, Characterization, and Encapsulation of Novel Plant Growth Regulators (PGRs) in Biopolymer Matrices

**DOI:** 10.3390/ijms22041847

**Published:** 2021-02-12

**Authors:** Kristina Vlahoviček-Kahlina, Slaven Jurić, Marijan Marijan, Botagoz Mutaliyeva, Svetlana V. Khalus, Alexander V. Prosyanik, Marko Vinceković

**Affiliations:** 1 Department of Chemistry, Faculty of Agriculture, University of Zagreb, Svetošimunska 25, 10000 Zagreb, Croatia; kvkahlina@agr.hr (K.V.-K.); sjuric@agr.hr (S.J.); marijan.marijan1986@gmail.com (M.M.); 2 Biotechnology Department, M. Auezov South-Kazakhstan University, Tauke-Khan, 160000 Shymkent, Kazakhstan; mbota@list.ru; 3 Ukrainian State University of Chemical Technology, Gagarina, 49000 Dnipro, Ukraine; swetasnegur9@gmail.com (S.V.K.); prosyanykav@gmail.com (A.V.P.)

**Keywords:** biopolymeric microcapsules, encapsulation, dehydroamino acids derivatives, plant growth regulators, agriculture

## Abstract

Novel plant growth regulators (PGRs) based on the derivatives of dehydroamino acids 2,3-dehydroaspartic acid dimethyl ester (PGR1), Z-isomer of the potassium salt of 2-amino-3-methoxycarbonylacrylic acid (PGR2) and 1-methyl-3-methylamino-maleimide (PGR3) have been synthesized and their growth-regulating properties investigated. Laboratory testing revealed their plant growth-regulating activity. PGR1 showing the most stimulating activity on all laboratory tested cultures were used in field experiments. Results showed that PGR1 is a highly effective environmentally friendly plant growth regulator with effects on different crops. Biopolymeric microcapsule formulations (chitosan/alginate microcapsule loaded with PGR) suitable for application in agriculture were prepared and characterized. Physicochemical properties and release profiles of PGRs from microcapsule formulations depend on the molecular interactions between microcapsule constituents including mainly electrostatic interactions and hydrogen bonds. The differences in the microcapsule formulations structure did not affect the mechanism of PGRs release which was identified as diffusion through microcapsules. The obtained results opened a perspective for the future use of microcapsule formulations as new promising agroformulations with a sustained and target release for plant growth regulation.

## 1. Introduction

The plant cells contain substances that are of great importance for the growth, development and functional activity of the whole organism. They are phytohormones—low molecular weight organic substances of endogenous origin. They are present in tissues in small quantities and with their help, the interaction of cells, tissues and organs is carried out. Phytohormones are required to start, regulate, turn on or turn off morphogenetic and physiological programs [1]. Phytohormones occur at low concentrations in plants and much research is focused on the preparation of synthetic hormonal products (plant growth regulators (PGRs)) suitable to affect plant growth and development in agricultural practice. They are used in fruit growing and horticulture, to combat lodging of cereals, to prevent the germination of tubers, root crops and bulbs during storage, to combat weeds, to accelerate the ripening of fruit, etc. The economic or agronomic benefits from the use of synthetic PGRs are many times higher than the costs made when studying the spectrum of the biological action of these physiologically active substances. The search for synthetic PGRs includes the synthesis of derivatives of various classes of organic compounds and testing their biological activities under conditions of vegetation and in field experiments. The list of such compounds is constantly growing. Due to the very high costs of development and registration, as well as the demand for a large market that offers high profitability, current research of synthetic PGRs is oriented toward application on globally important cereals such as maize, barley, wheat, etc. [2].

An analytical review of published data on PGRs showed that the promising class of compounds are activated amines containing carbonyl groups in the amine group (enamines), in particular, derivatives of dehydroamino acids [1,2,3,4,5,6,7]. They are synthetic analogs of a derivative of a natural amino acid that stimulates the production of the necessary phytohormones, as well as the rich source of nitrogen and universal highly effective environmentally friendly plant growth regulators that work on many crops.

We have synthesized novel PGRs, derivatives of dehydroamino acids, such as 2,3-dehydroaspartic acid dimethyl ester (Z-2-aminobut-2-endioate) and its monopotassium salt (potassium salt of Z-α-amino-β--methoxycarbonylacrylic acid), and 1-methyl-3-methylaminomaleinamide (1-methyl-3-methyl-aminopyrrol-2,5-dione) with various states of aggregation, possessing different lipophilicity, solubility in water, and biological activity to plants. Although PGRs have been developed to alter the physiology or growth of plants (to increase or decrease plant growth) there are certain limitations in their use; some of them are unstable when applied, phytotoxic, the method of application may result in significant material loss and can lead to the release of PGR into the environment, etc. To improve the economic or agronomic benefits of commercial crops, it is important to design a formulation containing PGR suitable for the application on mass-produced crops.

Encapsulation in microparticles is an advanced technology by which active agents are loaded into the carrier matrix. The benefits are: (i) active agents are protected from the environment, (ii) their degradation during the application decreases, (iii) lower active agent’s quantity is used and the most important fact is (iv) the possibility of active agent controlled delivery to the plants. Encapsulation in biopolymer matrices has been already recognized as an effective method for the controlled release of fertilizer [8]. Polysaccharides such as chitosan (CS) and alginate (ALG) are biopolymers that easily create microcapsules in which an active ingredient can be incorporated using an aqueous system at ambient temperature. There are many possible applications of chitosan/alginate microcapsules for immobilization and controlled release of various chemical or biologically active agents [9,10,11,12]. Through the process of ionic gelation and polyelectrolyte complexation, it is possible to prepare microcapsules simultaneously loaded with biological and chemical agents (macro-and micronutrients) which are essential for normal plant growth and survival. Calcium is an essential plant macronutrient and an integral part of plant cell wall structure involved in many processes occurring in plants [13,14]. Ca^2+^ is generally found in soil but it is relatively insoluble in the prevalent form. Since calcium is required in relatively large concentrations for normal cell growth, a sufficient amount of calcium must be always available in the root environment. Calcium alginate microparticles have been recognized as an effective formulation for controlled calcium release because, in the microbeads, calcium remains in the Ca^2+^ form, which is taken up by the plant through the root system [10].

We hypothesize that by encapsulation of synthesized PGRs in microcapsules an enhanced agroformulation with PGR as well as calcium delivery for the entire crop cycle can be prepared. This paper describes the synthesis and testing of the new compounds on maize, barley, wheat, and potatoes, as well as preparation and characterization of chitosan/alginate microcapsules loaded with PGR (CS/(ALG/(Ca+PGR)). The advantages of the new microcapsule formulations will be the protection of encapsulated PGR from degradation, reduced environmental impact and sustained release to regulate plant growth. The presence of a PGR and macronutrient calcium in novel microcapsule formulations could give wider opportunities for the application of different plant cultures.

## 2. Results and Discussion

The results are presented and discussed in two parts. In the first part, the plant growth-regulating activity of synthesized compounds (PGR1, PGR2, and PGR3) are assessed. In the second part, molecular interactions between microcapsule formulation constituents are analyzed and the essential physicochemical properties of microcapsule formulations without CS/(ALG/Ca) and with a PGR, CS/(ALG/(Ca+PGR)) discussed.

### 2.1. Assessment of Plant Growth-Regulating Activity of Synthesized PGRs

#### 2.1.1. Testing of Plant Growth-Regulating Activity

The results of testing plant growth-regulating activity are listed in Table 1.

The use of PGR1 in all tested concentrations accelerates plant growth, which is manifested in an increase in dry weight of seedlings, especially the mass of root systems. When PGR1 is used at a concentration of 0.001%, the plant root system and, to a lesser extent, the stem system for almost all studied cultures are at or above the standard level. This should positively affect the further growth of plants in adverse weather conditions, especially with a lack of moisture. Test compounds exert a significantly lesser effect on the development of the root system of barely and it is practically absent for wheat. The PGR2 test on maize and barley seeds was found to affect the growth of roots and stems of seedlings following the standard in all aspects, especially at the level of 0.01%. At the same time, PGR2 did not have a positive effect and even slightly inhibited the growth of wheat seedlings. In contrast, PGR3 promoted the growth of wheat seedlings, especially at a concentration of 0.0001% and, to a lesser extent, maize seedlings at a concentration of 0.01%, inhibiting the development of barley seedlings in all concentrations. Obtained data show a sharp dependence of the influence of the investigated compounds on the type of culture. The observed increase in the plant growth-regulating properties of the test compounds with a decrease in their concentration from 0.01% to 0.0001% solutions (inhibition at high concentrations—0.01%), significantly exceeded the standard. Based on the obtained data, it can be considered that the most promising plant growth regulator is PGR1 showing high stimulating activity on all tested crops, especially on the development of their root system.

#### 2.1.2. Testing of Auxin- and Gibberellin-Like Activity

Testing of auxin-like activity was performed by following the growth of segments of corn coleoptiles and mesocotyls in 24 h of exposure and the growth of whole coleoptiles in 24, 48 and 72 h of exposure to PGR solutions of different concentrations. During 24 h of incubation in water, 1 cm of corn coleoptiles grew by 2.3–3.5 mm. The impact of indole-3-acetic acid (IAA) 5 mg dm^−3^ stimulated their growth by 2.5 times to 7.3–7.9 mm. Such an increase in the lengths of coleoptiles in auxin is normal and confirms the high sensitivity of this test object to auxin. PGR1 inhibited the elongation of coleoptiles, showing a decrease in increments from 13 to 31% compared to the increment in water with increasing concentration from 0.1 to 100 mg dm^−3^. PGR2 was tested not only on the growth of one-centimeter segments of coleoptiles, but also on the growth of mesocotyl segments of corn seedlings, as well as on the growth of whole coleoptiles. The growth rate of one-centimeter segments of mesocotyls in the control variant was two times weaker than that for segments of coleoptiles. Specimen for PGR2 did not reveal a significant increase in the germination of the studied objects. There was no influence on the growth of the corn coleoptile segment at concentrations from 0.1 to 100 mg dm^−3^, but at lower concentrations (from 0.001 to 10 mg dm^−3^) PGR2 exhibited some auxin-like stimulating effect on the growth of corn coleoptile segments. The increase in mesocotyls under the influence of all PGR2 concentrations either did not exceed the control increase, or it is slightly lower than the control. At the same concentrations, PGR3 did not show an unambiguous effect on the growth of the test object, showing either a slight stimulation or a slight decrease in growth.

Testing of gibberellin-like activities of dehydroamino acid derivatives was performed using corn seedlings as test objects. Treatment of corn seedlings with gibberellic acid at a concentration of 10 mg dm^−3^ accelerates the growth of the first leaf of coleoptiles almost 1.5 times indicating a high sensitivity of test object to gibberellin. PGR1 used at a concentration of 0.1 mg dm^−3^ did not affect the leaf growth in 24 h of exposure, while PGR2 and PGR3 at the same concentration increased the leaf growth by 16 and 7%, respectively.

#### 2.1.3. Field Trials

The synthesized compounds showed distinct plant growth regulation activity. The most stimulating activity on all laboratory tested cultures showed PGR and this compound was applied in the field experiments Meteorological conditions of research during field trials are listed in Supplementary Tables S1 and S2. PGR1 was used by treating seeds (semi-dry treatment, inlay), tubers, cuttings, grafts, the root system of seedlings and vegetative plants. Field trials revealed a significant increase in the productivity of winter wheat (Supplementary Table S3) and barley (Supplementary Table S4). In the absence of pre-sowing treatment, spraying vegetative plants at the beginning of flowering increased the productivity of barley by 0.3 t ha^−1^ (9% control) and oats by 0.4 t ha^−1^ (8% control). In the latter case, the increase in productivity was facilitated by an increase in the productive grain of the broom (53% decrease in empty grain relative to control). Seed treatment before sowing with 0.0001% aqueous solution of PGR1 protected plants during the growing season. The artificial infestation of winter wheat plants during the flowering season by the Fusaria population did not lead to reduced yields. In contrast, it remained above the values observed for uninfected control plants (Supplementary Table S5). Treatment of potato tubers before planting with aqueous solutions of PGR1 led to a significant increase in yield (Supplementary Table S6). At the same time, the quality of tubers improved (Supplementary Table S7), the incidence of bacteriosis and viral diseases, late disease, fusarium wilt and nesting decreased (Supplementary Table S8). Field experiments have shown that the first treatment with PGR1—seeds before sowing, tubers before planting, cuttings when cutting and grafting, root systems of seedlings when sewing or planting—ensured early seed germination, good rooting, and increased plant resistance to adverse natural factors. Another treatment carried out in the budding phase and at the beginning of flowering revealed a decrease in ripening and an increase in harvest.

Based on the above data we can assume that PGR1 is a universally effective stimulator of plant development. Depending on the culture, time and method of processing, it increased germination and germination energy of seeds, accelerated the awakening and development of tubers, stimulated the formation of roots and blisters, improved photosynthesis by increasing the number of leaves and leaf surface of plants, increased crop yields, improved product quality, increased frost resistance and accelerated the regeneration of damaged plants, increases plant disease resistance and contributed to the safety of plant products during long-term storage. According to the principle of action, it is probably a promoter of endogenous phytohormone biosynthesis and/or an immunomodulator. In addition, PGR1 is environmentally friendly due to its low toxicity (LD_50_ 5163.4 mg kg^−1^) and extremely small application rate (10–300 mg) kg^−1^ (see details in Supplementary Information).

### 2.2. Physicochemical Properties of Microcapsule Formulations

#### 2.2.1. Molecular Interactions between Constituents in Microcapsule Formulations

Information on molecular interactions between PGRs, calcium chloride, chitosan and sodium alginate was obtained using FTIR. Spectra of single enamines and spectrum of their mixtures with calcium chloride, alginate or chitosan are presented and discussed in Figure S1a–d, respectively. Experimental vibrational frequencies and band assignments of PGRs are listed in Supplementary Table S9.

**Figure 1:**
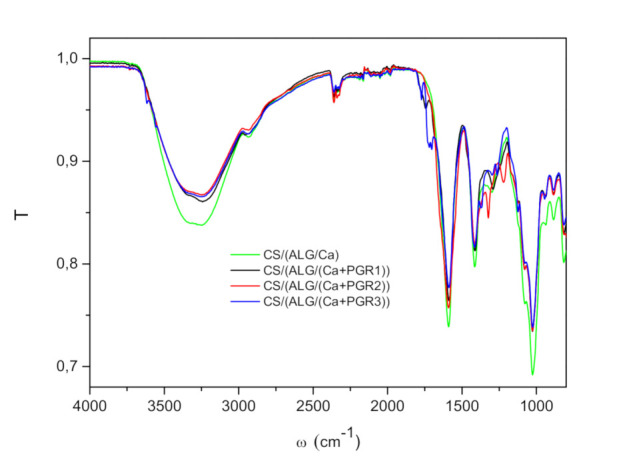
Characteristic FTIR spectra of dry microcapsule formulations; CS/(ALG/Ca) (green line); CS/(ALG/(Ca+PGR1)) (black line), CS/(ALG/(Ca+PGR2)) (red line) and CS/(ALG/(Ca+PGR3)) (blue line).

Spectra of microcapsules prepared without CS/(ALG/Ca) and with PGR CS/(ALG/(Ca+PGR)) are presented in Figure 1. All spectra exhibited bands of lower intensities in comparison with the CS/(ALG/Ca) spectrum. The bands corresponding to CS/(ALG/Ca) overlap those bands of microcapsule loaded with PGR. All spectra of CS/(ALG/(Ca+PGR)) exhibit changes of the strong and broad absorption band in the range 3000–3600 cm^−1^ (O–H and N-H stretchings) and asymmetric and symmetric stretching peaks of carboxylate (COO^−^) groups. The band intensity decreasing suggests a decrease in intermolecular hydrogen bonds which is connected with the presence of nitrogen atoms in encapsulated PGR.

A spectrum of the CS/(ALG/(Ca+PGR1)) shows a peak at approximately 1750 cm^−1^ due to the C=O stretching of the free carboxylic acid, alginic acid arising from hydrolysis of sodium alginate in the reaction vessel. This indicates that not all carboxylic acids will form a carboxylate with amine and some remain as free acids with a lower effect on the amino group [15]. New bands with a maximum at 1327 cm^−1^ and 1217 cm^−1^ corresponding to C-N stretching (aliphatic amines) appear in CS/(ALG/(Ca+PGR2)) spectrum. Also, new bands in the spectrum of CS/(ALG/(Ca+PGR3)) appear in the range of 1800 to 1600 cm^−1^; peaks at 1783 cm^−1^ and one wide peak with two peaks at 1730 and 1698 cm^−1^. These bands and peaks represent C=O stretching and secondary amine N-H band. Also, the appearance of a small peak at 1253 cm^−1^ represents the stretching of the C-N tertiary amines.

In all FTIR spectra peaks of chitosan and alginate, functional groups are somewhat shifted due to electrostatic interactions. Characteristic peaks of loaded and unloaded microcapsules are peaks at approximately 1420 cm^−1^ (C=O stretching band) and approximately 1610 cm^−1^ (carboxyl group) [16]. All shifts indicate complex electrostatic interactions and changes in hydrogen bonds [17]. Information about molecular interactions between microcapsule components and synthesized PGR revealed functional groups of all constituents interact with each other including mainly electrostatic interactions and hydrogen bonds.

#### 2.2.2. Morphology, Size, and Shape of Microcapsule Formulations

**Figure 2:**
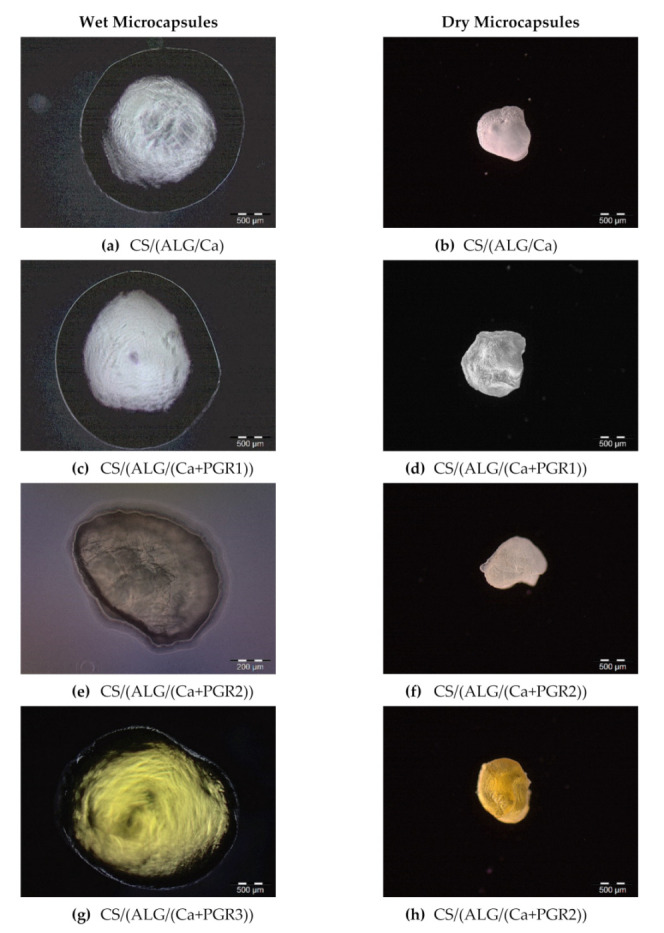
Optical microscope microphotographs of wet (left) and dry (right) microcapsules as denoted. Microcapsule was prepared without (**a**,**b**) and with PGR1 (**c**,**d**), PGR2 (**e**,**f**) and PGR3 (**g**,**h**). Bars are indicated.

**Figure 3:**
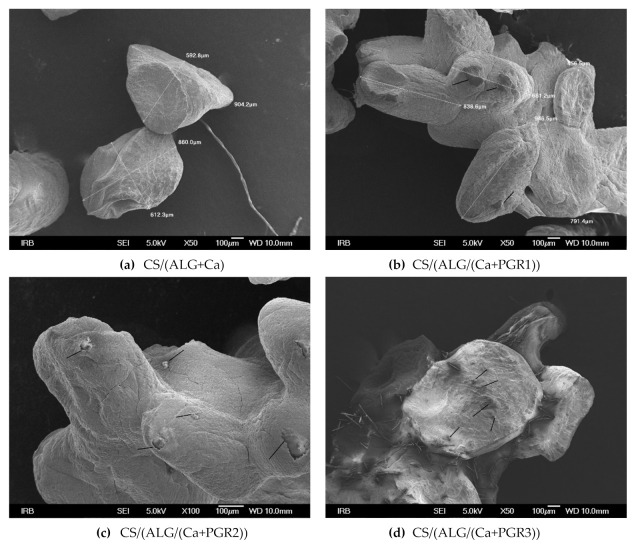
SEM microphotographs of dry microcapsule formulations (as denoted). Microcapsules were prepared without (**a**) and with PGR1 (**b**), PGR2 (**c**) and PGR3 (**d**). Aggregated nubs are marked by black lines (**c**,**d**). Bars are indicated.

The morphology, size and shape analysis of the wet and dry microcapsules were carried out by an optical microscope (Figure 2) immediately after the preparation and scanning electron microscopy (SEM) after drying to constant mass (approximately four weeks on air at room temperature) (Figure 3). All wet microcapsules were almost spherical, but after drying to constant mass, their sphericity was lost. The analysis of wet microcapsules showed that PGR encapsulation did not have significant effect on their size which ranged from 1706.07 to 2120.64 µm, respectively (CS/(ALG/Ca) = 2053 ± 114 µm; CS/(ALG/(Ca+PGR1)) = 2120.64 ± 173.30 µm; CS/(ALG/(Ca+PGR2)) = 1706.07 ± 175.51 µm; CS/(ALG/(Ca+PGR3)) = 2104.60 ± 188.23 µm).

The colors of prepared formulations are white (PGR1), light yellow (PGR2) and yellow (PGR3) as a consequence of entrapped PGR (Figure 2a,c,e,g). The surface of wet microcapsules was smooth, dense, and round. The smooth surface is a consequence of chitosan binding on sodium alginate chains [18]. Upon drying to constant mass, microcapsules lost their spherical shape and uniformity in size (Figure 2b,d,f,h). The surface of all dry microcapsule formulations was not smooth anymore, the wrinkles appeared and the surface became very rough. This could be explained by the partial collapse of the polymer network during dehydration [19]. Also, all prepared microcapsule formulations contained ~98% water. With the removal of water and with humidity loss, the size of all prepared microcapsules dramatically decreased by more than double which is associated with biopolymer strain-relaxation processes [20]. The size of dry microcapsules ranged from 745 to 810 µm (CS/(ALG/Ca) = 810 ± 94 µm, CS/(ALG/(Ca+PGR1)) = 806 ± 123 µm, CS/(ALG/(Ca+PGR2)) = 745 ± 121 µm, CS/(ALG/(Ca+PGR3)) = 797 ± 88 µm). Furthermore, we have observed more intense color change in dried microcapsules compared to the wet microcapsules due to the higher concentration of PGR, respectively to the mass.

SEM microphotographs of dried microcapsules are shown in Figure 3. The size of dry microcapsules observed by SEM follows values obtained by optical microscopy (OM).

After drying the spheric form of wet microcapsules was lost. Striped and fibrous surfaces representing the chitosan layer can be seen on CS/(ALG/Ca) surface (Figure 3a). In comparison with the microspheres, the microcapsules’ surface porosity was reduced [20]. Loading of CS/(ALG/Ca) with PGR resulted in the appearance of locally aggregated PGR (nubs) (aggregated nubs marked by black lines, Figure 3b–d). Energy dispersive X-ray (EDX) analysis showed that chemical compositions on these small nubs are equal to the chemical composition of PGR loaded into microcapsules. The surface of a microcapsule showed fibrous network propagation along the gel membrane surface [21]. It can be seen that some microcapsule surface parts exhibit the partial collapse of the polymer network as a result of drying.

#### 2.2.3. Encapsulation Efficiency, Loading Capacity and Swelling Degree of Microcapsule Formulations

Loading efficiency and loading capacity determination were performed to obtain information on the yield and the content of PGR in chitosan/alginate microcapsule. It is known that the entrapped amount of active agents in chitosan/alginate microcapsule depends on the type and concentration of both biopolymers, gelling cation and active agents, as well as the method of preparation [21,22]. Results listed in Table 2 show significant differences in both Encapsulation efficiency (EE) and loading capacity (LC) between samples. The difference in the EE reflects the extent of electrostatic interactions and hydrogen bonds involved in interactions of PGRs with both calcium chloride and sodium alginate during preparation. The most water-soluble PGR—PGR2 exhibited the highest EE and LC values indicating a greater extent of molecular interaction in solution. In addition to molecular interactions, the encapsulation capacity depends on the structure of the PGR, too. The smallest LC was determined for PGR3 which is a cyclic compound with a primary and tertiary amino group and two keto functional groups differing significantly in the structures from aliphatic primary amine (see details in Supplementary information).

When hydrophilic microcapsules are dispersed in the water they start to swell. This process starts with the penetration of the solution into the microcapsule through the surface and with polymer stress relaxation (transition of glassy structure to a rubbery state) [23]. One of the main factors which determine alginate swelling is a concentration of calcium ions which has got a major effect on the kinetics of gelation and the characteristics of the gel formed [24]. During gelation, calcium cations cooperatively interact with a block of L-guluronic acid groups forming ionic crosslinks between different polymer chains. The penetration of water into the denser network with high cross-linking density is difficult, i.e., swelling is limited by crosslinks and the S_w_ can be used as a measure of the extent of crosslinking [25].

A peculiar property of the chitosan/alginate microcapsules in the dry form is their ability, after contact with an aqueous fluid, to rehydrate, take up the fluid, and undergo a swelling process, mainly associated with the hydration of the hydrophilic groups of chitosan. When the fluid is water, it penetrates the microcapsules, fills the pores on the surface causing swelling without erosion/disintegration [26]. Chitosan/alginate microcapsules would interact with the water molecules through their available −COO^−^ and –NH_3_^+^ groups [27]. As can be seen in Table 2 all microcapsules show a relatively high degree of swelling which can be explained by the hydrophilic nature of the chitosan-alginate complex on the microcapsule surface [28]. The addition of PGR alters the number of alginate strands held together in the three-dimensional network and thus changes its strength and microcapsule structure. The highest swelling degree value obtained for PGR1 could be explained by less resistance against expansion and penetration of water molecules into its structure due to less crosslinking. Figure 3a,c revealed presence of an PGR on the microcapsule surface. It seems that the surface of microcapsules loaded with PGR1 contains the largest number of amino groups that would react with water contributing to a high swelling degree.

#### 2.2.4. Mechanisms and Kinetics of PGRs Release from Microcapsule Formulations

The release of active agents from hydrophilic microcapsules involves several different interconnected processes from the penetration of the surrounding solution into microcapsules, swelling, diffusion of the active agent through the gel membrane, dissolution of the active agent in the medium to the erosion of the swelled gel matrix. Kinetics and mechanism of active agent release primarily depend on the characteristics of the core material and active agent. In the case of a hydrophilic biopolymer matrix, active components are uniformly distributed/dissolved in the gel matrix and the release occurs by diffusion, swelling and matrix erosion [23]. The possible use of biopolymer microcapsule formulations loaded with PGR as a new potential fertilizer involves tunning and optimization of many characteristic parameters (preparation process, microcapsules chemical composition, geometry and size, the conditions during release, etc.) among which are the most important type and concentrations of biopolymer, gelling cation and active agents [29]. All the above parameters and their combination have an important influence on PGRs release from microcapsule formulations.

**Figure 4:**
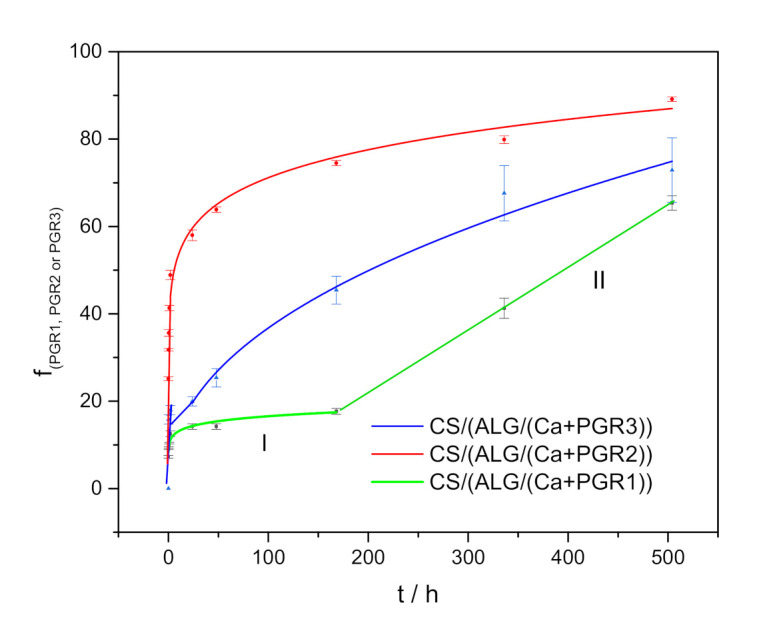
The fraction of released PGRs (f_(PGR1, PGR2, or PGR3)_ with time from microcapsule formulations CS/(ALG/(Ca+PGR)): I—the first time interval, II—the second time interval.

Microcapsule formulations are reservoirs of microscopic size surrounded by a wall that can control the release from the reservoir. When they are dispersed in water, it penetrates them, fills the pores among the polymer chains causing swelling. The release profiles of PGRs from microcapsule formulations with time are presented in Figure 4. All release profiles exhibit rapid initial PGR release followed by a slower release obeying the power-law equation. To explain and understand the kinetics and type of mechanism involved in the release, Korsmeyer-Peppa’s model was applied [30]. According to this model different controlling mechanisms may be distinguished by a simple empirical equation:

(1) $$f\;={\;kt}^{n}$$

where *k* is a release constant characteristic for a particular system considering structural and geometrical aspects, *n* is the release exponent representing the release mechanism, and *t* is the release time.

According to Korsmeyer–Peppas empirical model, the release exponent *n* can be characterized by three different mechanisms (Fickian diffusion, anomalous (non-Fickian diffusion), or Type II transport). Values of *n* < 0.43 indicates the release is controlled by classical Fickian diffusion, *n* > 0.85 is controlled by Type II transport, involving polymer swelling and relaxation of the polymeric matrix, whereas values of *n* between 0.43 and 0.85 show the anomalous transport kinetics determined by a combination of the two diffusion mechanisms and Type II transport.

The values of the release constants, exponents, and correlation coefficients of PGRs are listed in Table 3. Lower *n* values than 0.50 indicate that the release is controlled by a classical Fickian diffusion. In the case of the Fickian mechanism, the rate of PGRs diffusion is much less than that of polymer swelling and relaxation. This behavior induces the formation of a gradient of solvent penetration. It can be seen in Table 3 that the release rate of PGR2 is the fastest and of PGR3 is the slowest. This can be attributed to the ring structure of PGR3 molecule which slows down diffusion through the biopolymer matrix.

In the case of microcapsule formulations loaded with PGR1 two separate time regions in the release profile can be recognized. In the first time interval, the release profile is characterized by rapid initial release followed by slower release obeying power low, and in the second time interval, the release profile is characterized by linear release with time. The second part of the release curve can be easily described as zero-order kinetics, described with the equation [31]:

(2) $$f_{i}={\;K}_{0}t$$

where *f_i_* represents the fraction of active agent dissolved during the time *t* and *K_0_* is the zero-order rate constant and *t* is the time.

For zero-order kinetics, the release of PGR1 is only a function of time and the process takes place at a constant rate independent of PGR1 concentration. This phenomenon can be explained by the high degree of swelling (Table 2), which can prove to be a good property that will allow release for a long time. When chitosan coating is permeable to active agent and water, after swelling, the core becomes hydrated and PGR1 dissolves until it reaches its saturation concentration or solubility.

## 3. Materials and Methods

### 3.1. Materials

Low viscosity sodium alginate (CAS Number: 9005-38-3); Brookfield viscosity 4–12 cps (1% in H_2_O at 25 °C) was purchased from Sigma Aldrich (USA). High molecular weight chitosan (CAS Number: 9012-76-4, molecular weight: 310–375 kDa) was obtained from Sigma Aldrich (St. Louis, USA). A commercially available product calcium chloride, CaCl_2_ was used as a calcium donating substance (Kemika, Zagreb, Croatia). All other chemicals were of analytical grade and used as received without further purification. Derivatives of dehydroamino acids were synthesized in the laboratory of professor Alexander Prosyanik (Dnipro, Ukraine).

### 3.2. Synthesis of Plant Growth Regulators

Plant growth regulators, PGR1 (2,3-dehydroaspartic acid dimethyl ester), PGR2 (Z-isomer of the potassium salt of 2-amino-3-methoxycarbonylacrylic acid) and PGR3 (1-methyl-3-methylaminomaleinimide) are synthesized as follows:

PGR1: 0.1 mol of bromine is added to a mixture of 0.1 mol of maleic acid dimethyl ester and 0.2 g of boron trifluoride ether at a temperature of 90–100 °C. A reaction mass is maintained at this temperature for 8 h, cooled, a solution of 0.3 mol of ammonia is added in 100 mL of methanol. It was held for 12 h, methanol was removed under reduced pressure, the residue was extracted with toluene, and filtered. Toluene was removed from the filter under reduced pressure, and the residue was distilled in vacuo. Yield 62%, T_boil._ 76–78 °C/0.5 mm Hg, T_melt_. 32 °C.

PGR2: Solution of 0.13 mol of KOH in 10 mL of methanol was gradually added at cooling and stirring to a solution of 0.1 mol of 2,3-dehydroaspartic acid dimethyl ester in 50 mL of methanol, held for 5 h at 20 °C, the precipitate was filtered off, washed with 20 mL of methanol, in two portions 50 mL of ether, dried in a vacuum desiccator and crystallized from a mixture of isopropanol: water 10: 1. Yield 54%, T_melt_. 239–240 °C.

PGR3: Solution of 0.92 g (29.6 mmol) of methylamine in 5 mL of abs. methanol containing 167 mg (3.1 mmol) of sodium methylate was added to the solution of 2.45 g (14.1 mmol) of *N*-methyl-2,3-Dehydroaspartic acid dimethyl ester in 3 mL of abs. methanol is added, incubated for 2 days at 20 °C, cooled to 5 °C. The precipitate is filtered off, washed with abs. methanol; is crystallized from methanol. Yield 74%. T_melt._ 146–147 °C.

FTIR and NMR data of synthesized compounds are presented in Supplementary information Table S10A,B). 

### 3.3. Preparation of Microcapsule Formulations (CS/(ALG/(Ca+PGR))

Microcapsules loaded with PGR (microcapsule formulations) were prepared in the two-step process, by the ionic gelation and polyelectrolyte complexation at ambient temperature as described by Vinceković et al. [9]. In brief, each of the PGR (PGR1, PGR2 or PGR3) was dissolved into 100 mL of sodium alginate solution (1.5%) and homogenized by mixing (magnetic stirrer) for 60 min. The mixture (contains: 1% (*w*/*v*) of PGR1, 1% (*w*/*v*) of PGR2, and 0.5% (*w*/*v*) of PGR3) was dripped at the 30–40 mL min^−1^ flow into 100 mL of CaCl_2_ solution (2%) (contains: 1% (*w*/*v*) of dissolved PGR1, 1% (*w*/*v*) of PGR2, or 0.5% (*w*/*v*) of PGR3) through the encapsulator nozzle size of 1000 μm at 40 Hz vibration frequency and 20 mbar pressure (Encapsulator Büchi- B390, Bütchi Labortechnik AG, Flawil, Switzerland) under constant magnetic stirring. To promote gel strengthening, microspheres ALG/(Ca+PGR) were kept at room temperature for an additional 30 min. Excess CaCl_2_ was removed by washing the microspheres three times with distilled water. In the second stage, washed microspheres were dispersed in 50 mL chitosan solution (0.5% (*w*/*v*) CS in 1.0% (*v*/*v*) CH_3_COOH) under constant stirring (magnetic stirrer). The contact time between microspheres and chitosan solution was about 30 min to give chitosan time to form a layer around the microspheres and formation of microcapsules, CS/(ALG/(Ca+PGR)). Microcapsules were filtered, washed with deionized water. Several microcapsules were allowed to air-dry at room temperature to reach their equilibrium moisture content.

### 3.4. Assessment of Synthesized PGRs Plant Growth-Regulating Activity

Plant growth-regulating activities of synthesized compounds were assessed by laboratory and in field experiments.

#### 3.4.1. Testing of Plant Growth-Regulating Activity

Laboratory tests of corn (sort Odessa 100), barley (sort Zernogradsky) and winter wheat (sort Fedorovka) sowing properties were carried out by the method of germination in Petri dishes on filter paper [1] (see details in the Supplementary Information).

#### 3.4.2. Testing of Auxin- and Gibberellin-Like Activities

A corn coleoptile elongation test was used to assay for auxin-like activity and either cut of four-day-old corn seedlings with a coleoptile knot or dwarf pea seedlings are used for the gibberellin-like activity. Experimental details are presented in Supplementary information.

#### 3.4.3. Field Trials

PGR1 was used by treating seeds (semi-dry treatment, inlay), tubers, cuttings, grafts, the root systems of seedlings and vegetative plants for field trials. Experimental details and meteorological data (Supplementary Tables S1 and S2) are presented in Supplementary Information.

### 3.5. Fourier Transform Infrared Spectroscopy Analysis

The Fourier transform infrared spectroscopy spectra were recorded with the FTIR Instrument—Cary 660 FTIR (MIR system) spectrometer (Agilent Technologies, Santa Clara, CA, USA). Spectra of alginate, calcium chloride and PGRs (PGR1, PGR2 and PGR3) (see details in Supplementary Information; Figure S1a–d and Table S9) and microcapsules were analyzed. Spectral scanning was done in the range of 400–4000 cm^−1^.

### 3.6. Microscopic Observations

Microcapsules size, morphology and topography were analyzed by microscopic techniques: (i) optical microscopy (OM) (Leica MZ16a stereo-microscope, Leica Microsystems Ltd., Switzerland) and (ii) scanning electron microscopy (SEM) (FE-SEM, model JSM-7000 F, Jeol Ltd., Tokyo, Japan). The average diameter of wet and dry microparticles was determined by optical microscopy using Olympus Soft Imaging Solutions GmbH, version E_LCmicro_09Okt2009. Sixty microparticles were randomly selected from batches produced in triplicate, to determine the size distribution.

Microcapsule formulations for SEM analysis were put on the high-conductive graphite tape. FE-SEM was linked to an EDS/INCA 350 (energy dispersive X-ray analyzer) manufactured by Oxford Instruments Ltd. (High Wycombe, UK). The ImageJ software was used for the determination of the size of pores on a microparticle surface.

### 3.7. Encapsulation Efficiency, Loading Capacity and Swelling Degree

The encapsulation efficiency (EE) was determined from the total concentration of PGR (*c_tot_*) and content of PGR in dry microcapsules (*c_load_*) as was previously presented [11,32,33]. Encapsulation efficiency was calculated by the equation:

(3) $$\mathrm{EE}\;=\;\left(c_{load}/c_{tot}\right)\;\times \;100$$

where *c_load_* = *c_tot_* − *c_f_* and *c_f_* is the concentration of PGR in the filtrate.

The solution was vortexed again and left in the dark for 15 min. The concentration of PGR was determined spectrophotometrically (Shimadzu, UV-1700, Kyoto, Japan) at λ = 380 nm for PGR1 and PGR2 and at λ = 398 nm for PGR3.

The PGR content in microcapsules (PGR1, PGR2 or PGR3) was determined by dissolving 1 g microcapsules in 10 mL of a mixture of 16.80 g (0.2 mol dm^−3^) NaHCO_3_ and 17.65 g (0.06 mol dm^−3^) Na_3_C_6_H_5_O_7_ 2H_2_O at pH 8. A mixture of buffer and microcapsules was placed on a magnetic stirrer (IKA topolino) at 400 rpm until all microcapsules have completely dissolved. The obtained solution is filtered through double muslin, and the concentration of PGR in the filtrate was determined by UV-VIS spectrophotometer (Shimadzu, UV-1700, Kyoto, Japan).

The loading capacity (LC) expressed as mg of PGR per 1 g of microcapsules was calculated by the equation:

(4) $${\mathrm{LC}}_{\mathrm{PGR}}\;=\;\left(w_{PGR}/w_{c}\right)$$

where *w_PGR_* is the weight of loaded PGR and *w_c_* is the weight of the microspheres.

Swelling depends, among other things, on the property of the dissolving agent, therefore, to avoid the influence of electrolytes from buffer solutions, the degree of swelling (S_w_) was determined by dispersion of microcapsules in deionized water. Dry microcapsules (100 mg) were transferred into tubes and allowed to swell at room temperature for three hours in 10 mL of distilled water to strike a balance. The weight of the moist swollen microcapsules was determined by weighing after absorbing moisture from the surface of the microcapsule using filter paper. S_w_ was calculated using the equation:

(5) $$\mathrm{Sw}/\%\;=\;\left(\left(w_{t}-w_{0}\right)/w_{0}\right)$$

where *w_t_* is the weight of the swollen microcapsules and *w_0_* is the weight of the dry ones. All measurements were repeated three times, and the results are presented as the mean with the corresponding standard deviation.

### 3.8. Release Profiles of PGRs from Microcapsule Formulations

In vitro studies of PGRs release from CS/(ALG/(Ca+PGR)) were investigated by dispersion of microcapsules in deionized water and allowed to stand during the experiment, at room temperature. Samples were prepared by dispersing microcapsules (20 g/50 mL of PGR1 and PGR2 and 20 g/150 mL of PGR3 in deionized water). At appropriate intervals, the dispersion was stirred for 60 s, aliquots were taken and the concentration of PGR was determined spectrophotometrically at λ = 380 nm for PGR1 and PGR2 and at λ = 398 nm for PGR3. Results are presented as the fraction of released PGR using the equation:

(6) $$f\;=\;\left(R_{t}/R_{tot}\right)$$

where *f* represents the fraction of PGR released, *R_t_* is the amount of PGR released at time *t*, and *R_tot_* is the total amount of PGR loaded in microspheres.

### 3.9. Statistical Analysis

Microcapsules characterization experiments were carried out at room temperature in triplicate. The obtained data were analyzed with Microsoft Excel 2016. All data were shown as a mean value with standard deviation.

## 4. Concluding Remarks

Plant growth regulators are molecules widely applied in agriculture to control plant growth and development (increase crop yield and improve quality of agricultural products). We have synthesized new plant growth regulators based on the derivatives of dehydroamino acids (2,3-dehydroaspartic acid dimethyl ester (PGR1)**,** Z-isomer potassium salt of 2-amino-3-methoxycarbonyl acrylic acid (PGR2) or 1-methyl-3-ethylamino imine of maleic acid (PGR3)). Laboratory testing and in field experiments on several plant species from Ukraine (maize, barley, wheat, potato) revealed their potential as plant growth regulators.

PGR1 showing the most stimulating activity on all laboratory tested cultures was used in field experiments (treating seeds (semi-dry treatment, inlay), tubers, cuttings, grafts, the root system of seedlings and vegetative plants). The first treatment seeds before sowing, tubers before planting, cuttings at cutting and grafting, root systems of seedlings at quilting or planting provided early germination of seeds, good rooting, and increased resistance of plants to adverse natural factors. The second treatment carried out in the budding phase and at the beginning of flowering reduced the ripening time and increased the yield. The results revealed that PGR1 is a very effective ecological plant growth regulator (promoter of endogenous phytohormone biosynthesis, immunomodulator) acting on many crops.

Although there are a large number of studies on PGRs, their agricultural use currently represents a relatively small part of the agrochemical market [2]. Limitations ranged from a partial or complete reduction in bioactivity when exposed to field conditions to the manner and frequency of application. Microencapsulation is an advanced technology offers several advantages for delivering PGR to plants, but relatively few studies have focused on the use of agrochemical formulations with encapsulated PGR [34].

Calcium alginate microcapsules coated with a chitosan layer loaded with synthesized PGR were prepared as new microcapsule formulations for their improved delivery to plants. Information about molecular interactions between biopolymers and plant growth regulators obtained by FTIR spectroscopy revealed functional groups of all constituents interact with each other. A complex electrostatic interaction and hydrogen bonding between all constituents in microcapsule formulations as well as PGR molecular properties caused changes in microcapsule structure, and consequently, in physicochemical characteristics. The addition of PGRs alters the number of alginate strands held together in the three-dimensional gel network and thus the extent of crosslinks between polymer chains. The highest swelling rate value obtained on the microcapsule formulation loaded with PGR1 can be attributed to the less crosslinked polymer chains and the partial localization of PGR1 on the microcapsule surface.

Besides the stability of a microcapsule formulation against external parameters, such as time, temperature, shear, etc. as well as the application techniques, a very important aspect is the release kinetics of the active ingredient. All release profiles exhibit PGR rapid initial release followed by a slower release obeying the power-law equation. Differences in microcapsule structure due to PGRs encapsulation and surface properties do not affect the controlling release mechanism identified as Fickian diffusion. A particular feature of microcapsules loaded with PGR1 is that their core after initial swelling and release became hydrated and dissolved until PGR1 reached saturation concentration or solubility resulting in a linear release dependence over time. Gradual release in the first two hours (12%) followed by the linear release of up to 504 h (65%) allows sustained PGR1 release during plant growth, reducing the need for frequency of application in the field.

A better understanding of the structure/property relationship in microcapsule formulations and mechanisms controlling plant growth regulator release enhances the ability to control the release behavior and may aid in developing microparticles with specifically tailored properties.

With all these results, it seems that the new formulations of microcapsules with PGRs have great potential for application in the agricultural production of various crops. The advantages of using microcapsule formulations are in protecting the encapsulated PGR from degradation, reducing the amount required, and achieving controlled release to regulate plant growth for the entire crop cycle. Our future investigations will be oriented to testing microcapsules loaded with PGR1 in field experiments.

## Figures and Tables

**Table 1:** Growth-regulating activity testing of PGR *.

Concentration %	0.01		0.001		0.0001	
№ Compound	Root	Stem	Root	Stem	Root	Stem
The Length (Weight) of Plant Parts to Control (%)						
Maize						
PGR1	79/127	112/100	78/129	115/102	79/127	111/99
PGR2	121/164	129/131	100/148	109/117	97/140	114/108
PGR3	115/114	127/76	110/89	111/86	104/79	111/79
Humate	103/136	110/97	-	-	-	-
Barley						
PGR1	105/109	94/116	110/123	96/119	95/115	90/116
PGR2	100/121	97/111	100/125	103/101	92/112	100/112
PGR3	81/104	89/72	86/107	86/91	87/96	88/90
Humate	103/102	100/103	-	-	-	-
Wheat						
PGR1	102/121	104/119	125/132	107/122	119/126	104/118
PGR2	83/93	92/94	91/96	104/103	84/100	101/100
PGR3	94/116	110/122	105/118	107/118	119/137	109/123
Humate	111/134	106/118	-	-	-	-

* PGR1 (2,3-dehydroaspartic acid dimethyl ester), PGR2 (Z-isomer of the potassium salt of 2-amino-3-methoxycarbonylacrylic acid) and PGR3 (1-methyl-3-methylaminomaleinimide). The seeds grown or soaked in water were used as a control. A 2.5% sodium humate solution was used as a standard.

**Table 2:** Encapsulation efficiency (EE), loading capacity (LC) and swelling degree (S_w_) of CS/(ALG/(Ca+PGR)) microcapsule formulations.

PGR	EE/%	LC/mg g^−1^	S_w_/%
PGR1	53.31 ± 0.01	7.79 ± 0.08	27.76 ± 2.40
PGR2	89.94 ± 1.43	15.60 ± 0.09	1.76 ± 1.04
PGR3	75.73 ± 1.14	0.24 ± 0.00	1.05 ± 0.18

**Table 3:** Variation of the release constant (*k*/h), exponent (*n*) and correlation coefficient (R^2^) of PGR released from CS/(ALG/(Ca+PGR)) microcapsule formulations.

Microcapsule Formulations	k/h	n	R^2^
CS/(ALG/(Ca+PGR1))	10.36	0.104	0.991
CS/(ALG/(Ca+PGR2))	38.17	0.134	0.991
CS/(ALG/(Ca+PGR3))	4.84	0.441	0.992

## Data Availability

The data presented in this study are available in Supporting Information.

## Supplementary Materials

The following are available online at https://www.mdpi.com/1422-0067/22/4/1847/s1, Figure S1: FTIR spectrum of (a) PGR1 (black line), PGR2 (red line) and PGR3 (blue line), (b) mixtures with CaCl2, (c) mixtures with ALG (black line), (d) mixtures with CS. Added spectra of CaCl2, alginate (ALG) and chitosan (CS) are denoted with green lines; Table S1: Statistics of meteorological characteristics based on observations at the Nikopol weather station during 2018., Table S2: Monthly distribution of precipitation, average wind speeds, air humidity and atmospheric pressure during 2018., Table S3: The influence of PGR1 (2,3-dehydroaspartic acid dimethyl ester) on winter wheat productivity, Table S4: The influence of PGR1 (2,3-dehydroaspartic acid dimethyl ester) on barley productivity, Table S5: The influence of PGR1 (2,3-dehydroaspartic acid dimethyl ester) on the productivity of winter wheat after artificial infec-tion with Fusarias, Table S6: The influence of PGR1 (2,3-dehydroaspartic acid dimethyl ester) on potato productivity, Table S7: The effect of PGR1 (2,3-dehydroaspartic acid dimethyl ester) on the productivity and content of tubers of potato sort Nevskiy, Table S8: The effect of presowing treatment with PGR1 (2,3-dehydroaspartic acid dimethyl ester) on diseases potato sort Temp., Table S9: Experimental vibrational frequencies and band assignments for PGR1 (2,3-dehydroaspartic acid dimethyl ester), PGR2 (Z-isomer of the potassium salt of 2-amino-3-methoxycarbonylacrylic acid), and PGR3 (1-methyl-3-ethyl-amino imide of maleic acid), Table S10: (A) 1H NMR data of enamines (PGR1, 2,3-dehydroaspartic acid dimethyl ester), PGR2 (Z-isomer of the potassium salt of 2-amino-3-methoxycarbonylacrylic acid), and PGR3, 1-methyl-3-ethyl-amino imide of maleic acid) and (B) C/H/N Elemental analysis.

## Abbreviations

ALG	alginate
CS	chitosan
EDX	energy-dispersive X-ray
EE	encapsulation efficiency
FTIR	Fourier transform infrared spectroscopy
IAA	indole-3-acetic acid
LC	loading capacity
LD50	lethal dose, 50%
NMR	nuclear magnetic resonance
OM	optical microscopy
PGR	plant growth regulator
SEM	scanning electron microscopy
Sw	swelling degree
