# Supplementary material for: Synthesis, Characterization, and Encapsulation of Novel Plant Growth Regulators (PGRs) in Biopolymer Matrices
Source: Int J Mol Sci. 2021 Feb 12;22(4):1847. doi: 10.3390/ijms22041847 (PMC7918939; doi:10.3390/ijms22041847)
Supplement: Supplementary file 1 [file ijms-22-01847-s001.pdf]

International Journal of *Molecular Sciences*

MDPI

Article

Supporting Information

Synthesis, Characterization and Encapsulation of Novel Plant Growth Regulators (PGRs) in Biopolymer Matrices

Kristina Vlahoviček-Kahlina [1], Slaven Jurić [1], Marijan Marijan [1], Botagoz Mutaliyeva [2], Svetlana Khalus [3], Alexander Prosyanik [3], and Marko Vinceković [1,*]

[1] University of Zagreb, Faculty of Agriculture, Department of Chemistry; Svetošimunska 25, 10000 Zagreb, Croatia; kvkahlina@agr.hr (K.V.-K.); sjuric@agr.hr (S.J.); marijan.marijan1986@gmail.com (M.M.); mvincekovic@agr.hr (M.V.)

2 Biotechnology Department, M. Auezov South-Kazakhstan University, Shymkent, pr. Tauke-Khan; mbota@list.ru (B.M)

3 Ukrainian State University of Chemical Technology, Gagarina av., Dnipro, Ukraine; swetasnegur9@gmail.com (S.V.K), prosyanykav@gmail.com (A.V.P)

* Correspondence: mvincekovic@agr.hr ; Tel: + 385 1 239 3953

Keywords: biopolymeric microcapsules; encapsulation; dehydroamino acids derivatives; plant growth regulators; agriculture

Table of Content

Methods:

Testing of plant growth-regulating activity SI-2

Testing of auxin- and gibberellin-like activities SI-2

Toxicity determination SI-2

Field trials SI-2

Meteorological conditions of research during field trials SI-2

Fourier transform infrared spectroscopy and NMR analysis SI-4

Results and discussion:

Field trials SI-4

Molecular interactions between constituents in microcapsule formulations SI-6

NMR and C/H/N Elemental analysis SI-9

Methods:

Testing of plant growth-regulating activity. Laboratory tests of the sowing properties of corn (sort Odessa 100), barley (sort Zernogradsky), winter wheat (sort Fedorovka) seeds were carried out by the method of germination in Petri dishes on filter paper [1]. Seeds were germinated in enamines aqueous solutions at concentrations 0.01, 0.001 and 0.0001 % (w/v) During germination, the following regimes were maintained: for corn 25 °C, barley 20 °C, winter wheat 20 °C. 50 pcs of barley and winter wheat seeds and 30 pcs of corn seeds were placed in each germinator. Before seed hatching, the seedlings were covered with dark paper, and then germinated during the photoperiod night: day 8:16 and temperature 22-24 °C. Plant analysis was performed on the 14th day. The repetition of the experiment was fourfold.

Seed cultivation was carried out in 500 ml chemical glasses with agar-agar. Initially, a minimal amount of water was poured into the glass to avoid dilution, and then water was added to the initial mark. For the first six days, the seeds were in complete darkness at a temperature of 22 °C. Then the growth took place at a photoperiod of 6:18. Biometric analysis of plants was carried out on day 14.

Testing of auxin- and gibberellin-like activities. A simple and reliable method for determining the auxin-like activity of any compounds is the method of direct growth of segments of coleoptiles of etiolated seedlings of oats, wheat or corn with fertilized top. The coleoptile of cereal plants is very sensitive to exogenous indole-3-acetic acid (IAA), and therefore it is a classic object for biological tests for auxin. The increase in the length of the segment after 24 h in the dark was taken as a measure of auxin-like activity. Deionized water and IAA (5 mg dm^{-3}) were used as controls. The growth rate of oat coleoptiles correlates well with the level of diffuse IAA [2]. This test is practically insensitive to gibberellins and cytokinins [3].

As a highly specific test for gibberellin, either cuts of four-day-old corn seedlings with a coleoptile knot [4] or dwarf pea seedlings [5] are used. These segments were placed in Petri dishes with the test solutions and incubated for 48 or 72 hours in a dark thermostat at 26 °C. The gibberellic acid solution was used as a standard. The growth of the first leaf protruding above the cylinder of the coleoptile was measured.

Toxicity determination. Toxicity during intragastric exposure was determined after the method of Sanotykiy, 2013 [6]. Average lethal doses (LD_{50}) for intragastric intake were established in sexually mature animals: rats - males weighing 180-240 g, female rats weighing 180-220 g, mice - males weighing 18-22 g. The substance was injected in its native form intragastrically on an empty stomach using a metal probe in compliance with the relevant rules. In total, 80 rats and 30 mice were used in the experiments.

The values of the administered doses ranged from 2000 to 8000 mg kg^{-1}. The obtained LD_{50} values for male and female rats were 5163 ± 811 and 6282±420 m1 kg^{-1}, respectively. The intragastric administration of PGR1 to white mice showed the LD_{50} value was 5350 ± 737 mg kg^{-1}.

Field trials. A day before sowing 1 ton of corn, barley or wheat seeds were treated with 0.001% of an aqueous solution of PGR1 (20 L) and dried. Seeds treated with water served as control. The area of the plot was 10 m^2.

In the absence of pre-sowing treatment, an aqueous solution of PGR1 was sprayed on vegetative plants at the end of the harvest stage – the beginning of flowering with at a rate of 200 L ha^{-1} on barely (sort Prariya), oats (sort Skakun) and Spalchik rice.

Meteorological conditions of research during field trials. Meteorological characteristics based on observations at the Nikopol weather station (2018) are presented in Tables S1 and S2.

Table S1. Statistics of meteorological characteristics based on observations at the Nikopol weather station during 2018.

Indicators	Average ±statist.error	Median	Minimum	Maximum	CV (%)
Precipitation (mm)	0.90±0.15	0.00	0.00	18.00	308.39
Wind speed (m/s)	2.28±0.07	2.00	0.00	8.86	59.77
Temperature (ºC)	10.89±0.56	10.93	–17.72	30.28	97.96
T_{min} (ºC)	6.47±0.49	5.80	–21.30	22.20	146.02
T_{max} (ºC)	15.17±0.62	16.00	–15.10	37.80	78.51
Relative humidity (%)	69.12±0.91	69.57	27.75	100.00	25.08
Atmospheric pressure (hPa)	1014.67±0.40	1014.13	992.55	1047.33	0.75

Table S2. Monthly distribution of precipitation, average wind speeds, air humidity and atmospheric pressure during 2018.

Month	Precipitation (mm)	Average wind speeds (m/s)	Air humidity (%)	Atmospheric pressure (hPa)
January	21.40	3.59	78.60	1022.8
February	5.20	2.45	84.23	1022.3
March	7.40	3.50	61.91	1015.2
April	36.40	2.37	65.97	1015.4
May	47.20	2.00	68.94	1013.5
June	64.30	2.37	63.28	1012.1
July	10.20	2.42	53.96	1011.9
August	16.80	1.30	51.99	1006.3
September	31.00	2.09	56.23	1010.1
October	26.70	1.78	72.64	1015.9
November	15.60	1.65	83.14	1017.9
December	46.70	1.84	89.91	1013.0
Total	328.90	2.28	69.23	1014.7

Precipitation on the territory during all time were falling extremely unevenly. In 2018, the duration of the period without precipitation made up 264 days. The maximum daily amount of precipitation varied within 16-18 mm (Table S1). The intensity of precipitation varied throughout the year. The greatest amount of precipitation in the growing season was observed in June, and the least - in August (Table S2). Annual precipitation was mainly due to intense precipitation in April-June 149.7 (45.5% per annum).

The average annual temperature made up 10.89 ± 0.56 ºC. The temperature range for the study period made up from -21.3 to + 37.8 ºC. The minimum temperatures were observed in January-February, and the maximum in July-August. The greatest temperature fluctuations were observed in winter or spring. The autumn period is usually marked by a sharp and distinct drop in temperatures.

The winds were predominantly east and north-east.

The highest value of the average atmospheric pressure was observed in January-February, after which the pressure in March dropped rather sharply and reached a minimum in August.

Fourier transform infrared spectroscopy and NMR analysis. The Fourier transform infrared spectroscopy (FTIR) spectra were recorded with the FTIR Instrument - Cary 660 FTIR (MIR system) spectrometer (Agilent Technologies, USA). Single samples of alginate, calcium chloride and enamines (PGR1, PGR2 and PGR3) and their mixtures were analyzed. Spectral scanning was done in the range of 400-4000 cm^{-1}.

^{1}H NMR spectra of enamines were recorded on a Varian VXP-300 spectrometer (300 MHz, internal standard - Me_4Si), chemical shifts in δ-scale (ppm), coupling constants in Hz). Mass spectra were recorded on a VG-70EQ 770 mass spectrometer in FAB mode (FAB) and on Kratos MS 890 mass spectrometer electron impact mode (EI) and chemical ionization mode (CI), gas-reagent isobutane.

Results and discussion:

Field trials

Table S3. The influence of PGR1 (2,3-dehydroaspartic acid dimethyl ester) on winter wheat productivity

Variant	Concentration (%)	Productivity, t/ha (% to control)						
		Soft wheat			Durum wheat			
		Albatros	Skifyanka	Yuna	Kristal-2	Korund	Yasma	L.921H99
Control	Water	4.25	8.01	9.77	5.19	7.53	5.66	8.48
IAA	0.01	4.37[a] (102.8)	8.32 (103.9)	8.15 (83.4)	6.72 (129.5)	7.46 (99.1)	5.34 (94.3)	7.74 (91.3)
PGR1	0.001	4.70 (110.6)	10.36 (129.3)	10.82 (110.7)	6.11 (117.7)	8.34 (110.7)	7.41 (130.9)	9.90 (116.7)

[a]Humate, 2.5 %

Table S4. The influence of PGR1 (2,3-dehydroaspartic acid dimethyl ester) on barley productivity

Variant	Concentration (%)	Productivity, t/ha (% to control)				
		Spring barley				Winter barley
		Zernogradsky	Preriya	Nutans 518	Pallidum107	Siluet
Control	Water	3.75	3.28	4.50	6.05	4.03
Humate	2.5	3.67(97)	3.47(105.8)	4.6(102)	6.34(105)	4.06(101)
PGR1	0.001	4.12(110)[a]	3.49(106.4)	5.00(111)	7.20(119)	4.53(112.4)

[a] 0.01%

Table S5. The influence of PGR1 (2,3-dehydroaspartic acid dimethyl ester) on the productivity of winter wheat after artificial infection with Fusarias

Variant	Concentration (%)	Productivity, t/ha (%)			
		Fedorovka	Peresvet	Yubileynyy 75	Briz
Control	Water	4.5	4.39	4.7	5.05
PGR1	0.0001	4.75(106)	5.55(126)	5.6(119)	5.1(101)

Table S6. The influence of PGR1 (2,3-dehydroaspartic acid dimethyl ester) on potato productivity.

Variant	Concentration (%)	Productivity, t/ha (%)					
		Belorusskiy	Gatchinskiy	Lugovskoy	Polet	Temp	Nevskiy
Control		9.0	23.6	26.3	18.5	27.8	29.5
PGR1	0.001	9.7(107.8)	24.2(102.5)	30.5(108)	19.2(104)	30.6(110)	31(105)
PGR1	0.0001	12.2(135.5)	29.0(122.9)	34.9(122)	24.0(129)	33.7(121)	31.6(107)

Table S7. The effect of PGR1 (2,3-dehydroaspartic acid dimethyl ester) on the productivity and content of tubers of potato sort Nevskiy.

Variant	Concentration (%)	Tuber content				Productivity	
		nitrates, mg/kg	starch, %	vitamin C, mg %	solids, %	t/ha	%
Control	-	180.4	12.7	21.3	18.4	29.51	100
PGR1	0.01	121.2	13.8	23.4	19.5	33.14	112.8
PGR1	0.001	130	13.2	22.7	19.0	31.58	107.0

Table S8. The effect of presowing treatment with PGR1 (2,3-dehydroaspartic acid dimethyl ester) on diseases potato sort Temp.

**Variant**	**Concentration (%)**	**Plant disease (%)**				
		**During the growing season**		**During storage**		
		bacteriosis	viral diseases	late blight	late blight-fusarium rot	late blight-rubber rot
Control	Water	1.1	1.1	0	0.8	2.2
PGR1	0.0001	0.2	0.6	0.9	0	0.9
PGR1	0.001	0.2	0.8	0	0	0.4

Molecular interactions between constituents in microcapsule formulations

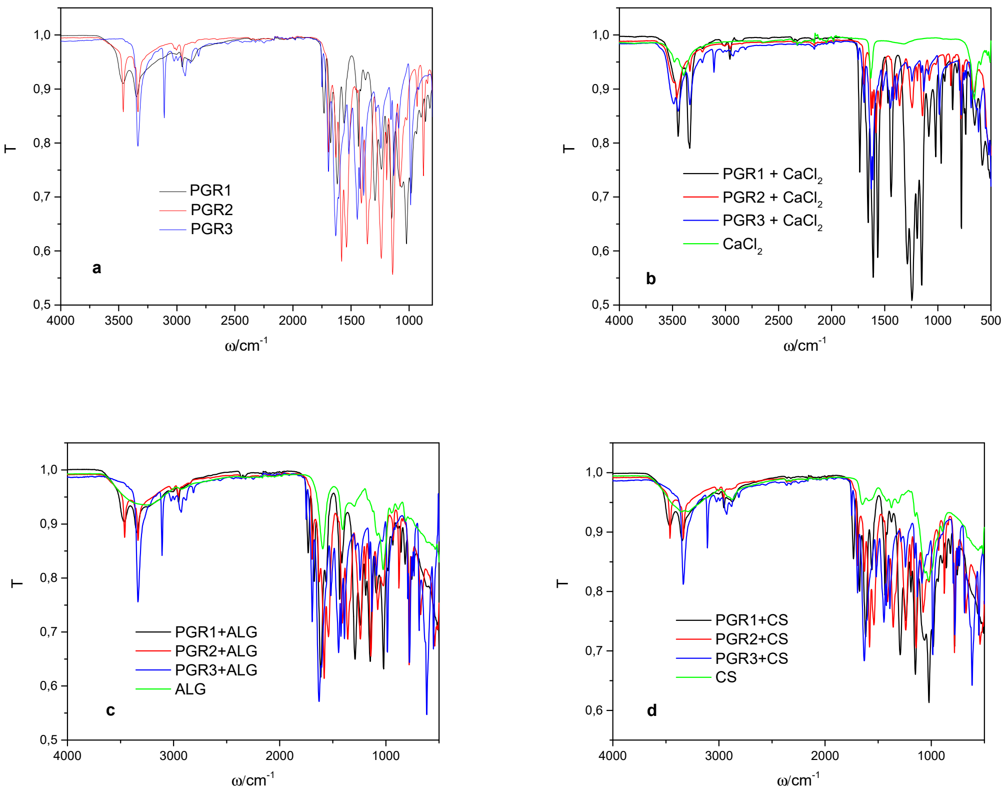

Figure S1. FTIR spectrum of (a) PGR1 (black line), PGR2 (red line) and PGR3 (blue line), (b) mixtures with $CaCl_2$, (c) mixtures with ALG (black line), (d) mixtures with CS. Added spectra of $CaCl_2$, alginate (ALG) and chitosan (CS) are denoted with green lines.

Information on molecular interactions between enamines, calcium chloride, chitosan and sodium alginate in mixtures was obtained using FTIR. Spectra of single enamines and spectrum of their mixtures with calcium chloride, alginate or

chitosan are presented in Figure S1a and S1b,c,d, respectively. Experimental vibrational frequencies and band assignments of enamines are listed in Table S9.

Spectra of PGR1 and PGR2 exhibit the spectral characteristics for aliphatic amines and spectrum of PGR3 for secondary and tertiary amines. Both, PGR1 and PGR2 spectra show the N-H stretching in the range of 3300-3000 cm^{-1} with two bands corresponding to asymmetric (higher frequency) and symmetric N-H stretching of primary amines. These bands are weaker and sharper than those alcoholic O-H stretching occurring in the same region. All characteristic peaks of PGR1 are relatively less intense than those of PGR2. PGR3 exhibits a sharp peak corresponding to secondary amine at 3332 cm^{-1}, and peaks characteristic for tertiary amine st 1240 and 1150 cm^{-1}.

Analysis of single calcium chloride, sodium alginate and chitosan spectra are previously reported [7]. The characteristic peaks in the calcium chloride spectrum are at 3494, 3396, 3214, 1646, 663 cm^{-1}. The frequency range 3214–3494 cm^{-1} and the medium intensity band at 1646 cm^{-1} represent the bending forms of hydroxyl groups. The medium intensity peak at 663 cm^{-1} represents the stretching of the Ca-O bond.

Analyses of sodium alginate and chitosan FTIR spectra were in correlation with literature data [8]. Characteristic vibration of sodium alginate spectrum is strong and broad absorption band in the range 3000-3600 cm^{-1} (O–H group), stretching vibration at 2920 cm^{-1} (the aliphatic C–H group), the bands at 1595 and 1405 cm^{-1} (asymmetric and symmetric stretching peaks of carboxylate ($–COO^-$) groups), weak broad stretching vibration at 1295 cm^{-1} (skeletal vibrations), and bands from 1081 to 1026 cm^{-1} (antisymmetric stretching (C-O-C).

Chitosan spectrum exhibits the strong and broad absorption band around 3330 cm^{-1} (O-H and -N-H stretching), stretching vibrations at 2925 cm^{-1} and 2875 cm^{-1} (asymmetric and symmetric modes of C-H), absorption band at 1648 cm^{-1} (amide I band), the bending vibrations at 1582 cm^{-1} (N-H stretching of N-from amine and amide II), band at 1425 cm^{-1} (CH_2 scissoring), medium peak at 1373 cm^{-1} (symmetrical CH_3 deformation), whereas vibrations in the 1190-920 cm^{-1} region represent C-N stretching vibration and overlap of the vibrations from the carbohydrate ring.

The FTIR spectra of a mixture of calcium chloride and enamines show differences in the position and intensity of the bands in comparison with single components due to enhanced hydrogen bonds and electrostatic interaction between calcium and amino groups (Figure 1b). The spectra with PGR1 show relatively higher intensity in comparison with spectra of PGR2 in the region of asymmetrical and symmetrical N–H stretching and N–H bend. There is the propagation of bands in the range of the wavenumbers from 3600 to 3200 cm^{-1} for mixtures with PGR1 and PGR2. There is a change in the position, shape and intensity of the bands corresponding to the primary amines: the bands move toward lower values of wave numbers: from 3473 to 3469 cm^{-1}, 3347 to 3334 cm^{-1}, 2960 to 2953 cm^{-1}, 1680 to 1653 cm^{-1}, 1617 to 1604 cm^{-1}, 1562 to 1568 cm^{-1}. The following bands also change: from 1238 to 1245 cm^{-1}, 1187 to 1192 cm^{-1}, 1148 to 1152 cm^{-1}, 1020 to 1018 cm^{-1}, 778 to 776 cm^{-1}. Changes in the position and relative intensities can be explained by the coordination and formation of bonds between calcium ions and the amino group, creating a $Ca(NH_3)_nCl_2$ type complex [9].

Spectra of enamines and ALG mixture presented in Figure 1c shows enhanced stretching vibrations in the range of the asymmetric and symmetric N-H stretching of primary amines (PGR1 and PGR2) and N-H stretching at 3288 cm^{-1} of secondary amine (PGR3). These changes can be explained by the enhanced hydrogen bonding. All other bands indicate the presence of amino groups, and the interactions are evident in the intensity decrease of bands. Enhanced intensities and shifting of bands corresponding to asymmetric and symmetric carboxyl groups (COO^-) higher wavenumbers observed for all samples indicated interactions with N-H groups. Changes in the position and intensity of the bands corresponding to the C=C and C-N stretching and as well as N-H wag occur.

Spectra of enamines with chitosan exhibit almost no shifting of characteristic bands but all characteristic peak intensities are enhanced due to reaction between the amino group of dimethyl esters of amino fumaric acid and the -OH group of chitosan as a result of different dipole moments of the groups, and the formation of saturated amides (Figure 1d). The peak intensities increase is significant in the PGR1 spectrum and much smaller in the PGR2 and PGR3 spectra.

FTIR analyses revealed complex interactions between enamines in mixtures with calcium chloride, sodium alginate or chitosan due to electrostatic interactions and strong hydrogen bonding.

Table S9. Experimental vibrational frequencies and band assignments for PGR1 (2,3-dehydroaspartic acid dimethyl ester), PGR2 (Z-isomer of the potassium salt of 2-amino-3-methoxycarbonylacrylic acid), and PGR3 (1-methyl-3-ethyl-amino imide of maleic acid).

Frequency/cm^{-1}	Comments
**PGR1**	
3600-3300	N-H stretching (primary amines)
3471	asymmetrical N–H stretching
3346	symmetrical N–H stretching
1700	C=O stretching
1670 and 1616	N–H bend (primary amines)
1438	C=C
1281	C-N stretchinging
1250 and 1190	C-N stretchinging (aliphatic amines)
910-665	N–H wag (primary amines)
**PGR2**	
3600-3300	N-H stretching of primary amines (appear as two peaks)
3473	asymmetrical N–H stretching
3347	symmetrical N–H stretching
1700	C=O stretching
1629 and 1580	N–H bend (primary amines)
1530	C=C stretching
1408	C-C stretching
1240 and 1193	C-N stretching (aliphatic amines)
910-665	N–H wag (primary amines)
**PGR3**	
3288	N-H stretching (secondary amine)
1700	C=O stretching
1650	N-H bend (secondary amines)
1438	C=C
1240 and 1150	C-N (tertiary amines)
1190 and 1130	N-H bend, C–N stretching (secondary amines)
1143	C-N stretching
763	N–H wag (secondary amines)

NMR and C/H/N Elemental analysis

Table S10. (A) ^{1}H NMR data of enamines (PGR1, 2,3-dehydroaspartic acid dimethyl ester), PGR2 (Z-isomer of the potassium salt of 2-amino-3-methoxycarbonylacrylic acid), and PGR3, 1-methyl-3-ethyl-amino imide of maleic acid) and (B) C/H/N Elemental analysis.

**(A)**	**^{1}H NMR (200 MHz, 25 °C)**				
**PGR1 (CDCl$_3$ from HMDS)**		**PGR2 (CD$_3$OD from HMDS)**		**PAGR3 (CDCl$_3$ from TMS)**	
δ / ppm	ASSIGNMENT	δ / ppm	ASSIGNMENT	δ / ppm	ASSIGNMENT
3.72	3H, s, CH$_3$O	3.62	3H, s, CH$_3$O	2.91	3H, d, CH$_3$NH
3.87	3H, s, CH$_3$O	4.92	1H, s, CH	2.95	3H, s, CH$_3$N
5.51	1H, s, CH			4.80	1H, s, CH
5.93	1H, s, NH				
7.76	1H, s, NH				

	**(B)**	**C/H/N Elemental analysis**				
	**PGR1: C$_6$H$_9$NO$_4$**		**PGR2: C$_5$H$_6$KNO$_4$**		**PGR3: C$_6$H$_8$N$_2$O$_2$**	
	FOUND %	CALCULATED %	FOUND %	CALCULATED %	FOUND %	CALCULATED %
**C**	45.42	45.28	32.96	32.78	51.69	51.42
**H**	5.53	5.70	3.06	3.30	5.37	5.75
**N**	8.74	8.80	7.33	7.65	19.99	19.99

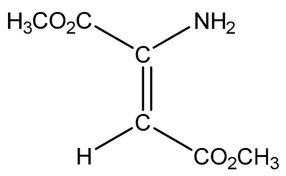

1
Chemical Formula: $C_6H_9NO_4$
Molecular Weight: 159,14

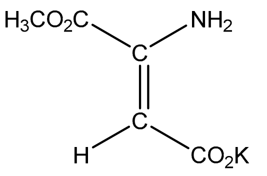

2
Chemical Formula: $C_5H_6KNO_4$
Molecular Weight: 183,20

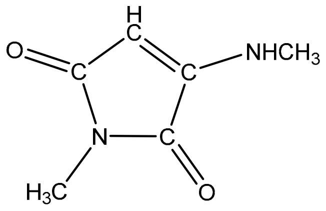

3
Chemical Formula: $C_6H_8N_2O_2$
Molecular Weight: 140,14

References

1. Khokhlova, T.V.; Chertikhina, Y.A.; Mutaliyeva, B.Z.; Kudasova, D.E.; Yanova, K.V. Aminofimaric acid derivatives: synthesis and influence on plant development. *Vopr. Khim. Khim. Tekhnol.* **2018**, *6*, 91–98.

2. Weiler, E.W.; Jourdan, P.S.; Conrad, W. Levels of indole-3-acetic acid in intact and decapitated coleoptiles as determined by a specific and highly sensitive solid-phase enzyme immunoassay. *Planta* **1981**, *153*, 561–571, doi:10.1007/BF00385542.

3. Kepheli, V.I.; Turetskaya, R.X.; Koph, E.P.; Vlasov, P.V. Determination of the biological activity of free auxins and growth inhibitors in plant material: Methods for the determination of phytohormones, growth inhibitors,

defoliants and herbicides. *M. Sci.* **1973**, 7–21.

4. Boyarkin, A.M.; Dmitriyeva, M.I. Methods for the determination of growth regulators and herbicides. *M. Sci.* **1966**, 12–23.

5. Lozhnikov, V.N.; Khlopenkova, L.P.; Chailyakhyan, M.X. Determination of natural hiberlins in plant tissues: Methods for the determination of phytohormones, growth inhibitors, defoliants and herbicides. *M. Sci.* **1973**, 50–58.

6. Sanotskiy, I.V. *Methods for determining the toxicity and hazard of chemicals*; 2013;

7. Stage, R.B.; Stanley, J.B.; Moseley, P.B. Determination of primary and secondary fatty aliphatic amines in the presence of large amounts of nitrile by near-infrared spectroscopy. *J. Am. Oil Chem. Soc.* **1972**, *49*, 87–89, doi:10.1007/BF02612632.

8. Jurić, S.; Đermić, E.; Topolovec-Pintarić, S.; Bedek, M.; Vinceković, M. Physicochemical properties and release characteristics of calcium alginate microspheres loaded with Trichoderma viride spores. *J. Integr. Agric.* **2019**, *18*, 2534–2548, doi:10.1016/S2095-3119(19)62634-1.

9. Schmidt, J.; Marcovitch, O.; Lubezky, A.; Kozirovski, Y.; Folman, M. IR and FIR spectra of ammonia adsorbed on calcium chloride and bromide: Evidence for complex formation. *J. Colloid Interface Sci.* **1980**, *75*, 85–94, doi:10.1016/0021-9797(80)90352-5.
